# Identifying how GPs spend their time and the obstacles they face: a mixed-methods study

**DOI:** 10.3399/BJGP.2021.0357

**Published:** 2021-11-30

**Authors:** Carol Sinnott, Jordan M Moxey, Sonja Marjanovic, Brandi Leach, Lucy Hocking, Sarah Ball, Alexandros Georgiadis, Guillaume Lamé, Janet Willars, Mary Dixon-Woods

**Affiliations:** THIS Institute (The Healthcare Improvement Studies Institute), Department of Public Health and Primary Care, University of Cambridge, Cambridge, UK.; THIS Institute (The Healthcare Improvement Studies Institute), Department of Public Health and Primary Care, University of Cambridge, Cambridge, UK.; RAND Europe, Cambridge, UK.; RAND Europe, Cambridge, UK.; RAND Europe, Cambridge, UK.; RAND Europe, Cambridge, UK.; THIS Institute (The Healthcare Improvement Studies Institute), Department of Public Health and Primary Care, University of Cambridge, Cambridge, UK.; THIS Institute (The Healthcare Improvement Studies Institute), Department of Public Health and Primary Care, University of Cambridge, Cambridge, UK; Laboratoire de Génie Industriel, CentraleSupélec, Université Paris-Saclay, France.; Department of Health Sciences, University of Leicester, Leicester, UK.; THIS Institute (The Healthcare Improvement Studies Institute), Department of Public Health and Primary Care, University of Cambridge, Cambridge, UK.

**Keywords:** ethnography, general practice, mixed methods, operational failures, time and motion studies, operations research

## Abstract

**Background:**

Although problems that impair task completion — known as operational failures — are an important focus of concern in primary care, they have remained little studied.

**Aim:**

To quantify the time GPs spend on different activities during clinical sessions; to identify the number of operational failures they encounter; and to characterise the nature of operational failures and their impact for GPs.

**Design and setting:**

Mixed-method triangulation study with 61 GPs in 28 NHS general practices in England from December 2018 to December 2019.

**Method:**

Time–motion methods, ethnographic observations, and interviews were used.

**Results:**

Time–motion data on 7679 GP tasks during 238 hours of practice in 61 clinical sessions suggested that operational failures were responsible for around 5.0% (95% confidence interval [CI] = 4.5% to 5.4%) of all tasks undertaken by GPs and accounted for 3.9% (95% CI = 3.2% to 4.5%) of clinical time. However, qualitative data showed that time–motion methods, which depend on pre-programmed categories, substantially underestimated operational failures. Qualitative data also enabled further characterisation of operational failures, extending beyond those measured directly in the time–motion data (for example, interruptions, deficits in equipment/supplies, and technology) to include problems linked to GPs’ coordination role and weaknesses in work systems and processes. The impacts of operational failures were highly consequential for GPs’ experiences of work.

**Conclusion:**

GPs experience frequent operational failures, disrupting patient care, impairing experiences of work, and imposing burden in an already pressurised system. This better understanding of the nature and impact of operational failures allows for identification of targets for improvement and indicates the need for coordinated action to support GPs.

## INTRODUCTION

Pressures on primary care are increasingly leading to difficulties in recruitment and retention of GPs.^1^^,^^2^ High levels of stress and burnout affect those who stay.^3^^–^^6^ Although the operational burdens of general practice are among the most important contributors to job dissatisfaction and stress,^7^^–^^9^ they have remained little studied,^10^^–^^12^ frustrating attempts to develop effective solutions. In contrast, a substantial literature has examined the challenge of operational failures in secondary care,^13^ where they are defined as problems in work-system design that impair workers’ effectiveness. In hospital settings, operational failures (including errors or defects in the flow of work, missing equipment or information, and interruptions that interfere with task completion^14^^–^^16^) are known to damage individual and organisational performance, consuming as much as 9% of nursing time in secondary care.^14^^,^^16^^–^^18^

A recent interview study^19^ identified concern among NHS GPs about operational failures in primary care, but the extent and impact of these failures and their nature is poorly understood. Direct observations of how GPs spend the time allocated to clinical care, and what might disrupt their ability to complete tasks, have remained remarkably rare.^12^ This article, using mixed methods, sought to address this void in the literature. The aims were to quantify the amount of time GPs spend on activities during clinical sessions, to identify the number of operational failures they encounter, and to characterise the nature of these operational failures and their impact for GPs.

## METHOD

A mixed-method triangulation study^20^ was conducted involving:

- time–motion methods to quantify the time spent on GP tasks and disruptions to tasks;
- ethnographic observations to characterise the nature and impact of operational failures; and
- interviews to understand what was observed from the perspective of GPs.

GPs across four clinical commissioning groups were invited to participate via the National Institute for Health Research Clinical Research Network.

Sample size was kept under review as the study progressed; observations of 61 GPs working in 28 different general practices were deemed to offer sufficient diversity and information power.^21^ Observations covering a clinical session (half day) were scheduled for each participating GP. For each session, the observer (one of three non-clinical researchers, who have backgrounds in engineering, psychology, and social science) collected time–motion data, made detailed ethnographic fieldnotes, and interviewed the observed GP.

**Table:** How this fits in

Direct observations of what consumes GPs’ time and what might disrupt their ability to complete tasks have remained remarkably rare. Operational failures are common in general practice and highly consequential. Frequent operational failures include interruptions interfering with task completion, problems relating to equipment and supplies, problems arising from GPs’ coordination role, and defects in organisational processes within practices. The impact of operational failures in general practice goes well beyond diversion of time and interference with task completion: they are very adverse for GPs’ experiences of work. This study, by providing a better understanding of the nature and impact of operational failures, helps identify targets for improvement and indicates the need for coordinated action to support GPs.

### Time-motion data and analysis

Defined as *‘the observation and analysis of movements in a task with an emphasis on the amount of time required to perform the task’*,^22^ time-motion studies are increasingly well-established techniques for describing the work done in healthcare environments.^23^ For this study, trained observers used a handheld computer device with customised software (The Work Observation Method by Activity Timing — WOMBAT^24^). This software has been shown to produce reliable and valid data on clinicians’ patterns of work, but has been used mainly in secondary care.^24^ To capture data specific to GPs’ work, the authors of this study developed six primary task categories and associated subcategories, based on classifications previously used in studies in US ambulatory care (Box 1).^25^^,^^26^ The categorisation was modified slightly after the first six observations. The categories were not mutually exclusive; observers could assign the same task to several categories/subcategories.

**Box 1.** Task categories pre-programmed into Work Observation Method by Activity Timing (WOMBAT) time-motion software^24^ and used by observers to classify observed activities

**Primary task categories (at least one option mandatory, multiple options possible)**	**Definition**	**Subgroup activity (at least one option mandatory if associated main activity chosen, multiple options possible)**
**Direct care**	Patient care/clinical work that GPs do for a patient when that patient is in the consultation room or on the phone	Preparing to see patient(s) Calling in patient History Examination Prescribing Documenting/updating electronic health record Explaining/two-way planning Telephoning Telephone consultation Procedure Psychosocial discussion Order bloods/do referral letters
**Clinical paperwork**	Patient care/clinical work that GPs do when they are not in direct contact with a patient	Reviewing bloods Prescribing Writing letters Reading letters Checking emails Actioning tasks Chasing up test results/letters
**Interactions with colleagues**	In-person communication with colleagues in the practice	About patients About processes About other
**Internet use**	GP uses internet to seek work-related information to facilitate their own work or support a consultation with a patient	Seeking information for referral, such as the GP looking up information on local services, or other internet searching to meet their own educational needs Supporting consultation such as signposting the patient to local services, showing patient websites or images related to their condition
**Operational failures and interruptions**	Problems in work-system design that resulted in GPs being less effective than they otherwise might have been, including problems in the supply of information, equipment, or materials to GPs, or situations where an interruption interfered with a completion of a task	Unexpected incoming phone calls Looking for missing equipment/materials Technology problems People coming into the consultation room unasked Requiring input from other members of the practice team to solve problems in task completion
**Home visits**	Home visits out of surgery to a patient’s home	Not applicable (note: in almost all observations, home visits were not observed or recorded in time–motion software)

On the agreed day, the observer shadowed the GP participant for an entire clinical session, recording and timestamping all the GP’s work activities using the software. Multitasking, defined as the conduct of ≥2 tasks simultaneously, was also captured.

Descriptive analyses were performed for: the total number of tasks; the total time that tasks were being actively performed in each primary task category; the proportion of time spent on various task categories; the frequency of and time spent multitasking; and the task categories interrupted by operational failures.

Linear regression was used to assess the relationship between the operational failure rate and practice size (indicated by number of GPs and patients). Student’s *t*-test was used to compare the rate of operational failures and GP sex, practice electronic health record (EMIS or SystmOne), session type (duty-doctor or routine), and whether the practice was part of an alliance (a group of general practices working as a single organisational entity).

Data were analysed in Microsoft Excel (2016) and Stata/IC (version 12.1). All analyses were adjusted for clustering by practice, and 95% confidence intervals (CIs) were calculated using normal approximation. The characteristics of participating GPs’ practices were retrieved from the National General Practice Profiles.^27^

### Qualitative data and analysis

Ethnographic observations were conducted that focused on how GPs were disrupted in their work and the immediate and general contexts relevant to those disruptions. Observers took written notes during the observations and subsequently prepared detailed fieldnotes by audio-recording themselves.

Semi-structured interviews were conducted using an interview guide (see Supplementary Figure S1) to explore the disruptions observed and GPs’ perceptions of these occurrences immediately after the observation session, or the following day by phone.

The fieldnotes and interviews were transcribed verbatim and anonymised. To facilitate initial organisation of the data, a framework was used based on findings from an interpretive review^12^ before proceeding to inductive analysis,^28^ with key themes identified through repeated close readings of the data. The themes generated were also compared with the categories used in the time–motion data collection. NVivo (version 12) supported the management of qualitative data.

### Consent

Written consent was obtained from GP participants. Patients were informed of the study by practice staff when booking their appointment, again when checking in for their appointment, and via waiting-room posters. Patients were assured that the observer was not collecting any personally identifiable patient information, and should they wish, would leave the room during their consultation.

Participating GPs repeated this information when calling patients into their consultations, and obtained patient consent verbally. Consultations where the observer was not present owing to patients declining were recorded as ‘direct patient care’ in the time–motion software.

## RESULTS

Sixty-one GPs (32 male and 29 female) working at 28 general practices providing care to over 298 000 patients participated in the study, which took place from December 2018 to December 2019. Practices were located in the East of England (Cambridgeshire *n* = 11, Peterborough *n* = 8, Hertfordshire *n* = 2, Bedfordshire *n* = 3, and Norfolk *n* = 4).

The practice sample was comparable with the English average for number of full-time GPs, number of registered patients, proportion of patients aged >65 years, proportion of patients with at least one long-term condition, deprivation decile, and deprivation score (Table 1).^27^^,^^29^ All GPs used electronic health records (EMIS *n* = 9, SystmOne *n* = 52), and 12 practices were part of an alliance.

**Table 1.** Characteristics of the practices of the participating GPs

**Characteristic**	**Median (IQR)**	**England average^27^^,^^29^**
Full-time equivalent GPs, *n*	5.5 (4.0–7.0)	5.16
Registered patients, *n*	10 671 (7772–12 576)	8852
Proportion of patients aged >65 years	16.9 (15.4–20.1)	17.5
Proportion of patients with at least one long-term condition	57.3 (47.6–59.9)	52.4
Deprivation decile	7.5 (4.0–10)	–
Deprivation score^a^	15.6 (9.9–27.9)	21.7

a *The larger the score, the more deprived the area. IQR = interquartile range.*

Across the 61 sessions observed, data was collected on 238 hours (h) 4 minutes (min) of GP work, with a median observation time per GP of 3 h 58 min (interquartile range [IQR] 3 h 16 min–4 h 27 min). Observations generally took place within the official start and end times of clinical sessions, so tasks done when GPs came in early (for example, paperwork undertaken before appointments began), worked over lunch, or took work home were not captured. Although ‘home visits’ was a pre-programmed category, visits were not observed directly, instead start and finish times only were noted.

Ethnographic data were collected for all 61 observations. Post-observation interviews were conducted with 51 GPs (total 19 h 57 min, median 23 min, IQR 17 min–29 min); 10 GPs were not available for interview. Each observer recorded a roughly equal number of observations, with no significant differences in number recorded between them.

### Time–motion data on how GPs spend their time in clinical sessions and what disrupts them

The time–motion study captured data on 7679 tasks undertaken by GPs (Table 2). Of these, 80.9%, (*n* = 6214, 95% CI = 80.0% to 81.8%) concerned direct patient care (for example, face-to-face and phone consultations). These tasks were responsible for 72.4% (172 h 24 min 14 seconds (s), 95% CI = 70.2% to 74.6%) of GPs’ time during the observations. Clinical paperwork (for example, reviewing blood test results, issuing prescriptions, writing referral letters, reading incoming letters, and actioning tasks related to patient care) accounted for 10.0% (*n* = 767, 95% CI = 9.3% to 10.7%) of GPs’ tasks, consuming 12.8% (95% CI = 11.4% to 14.2%) of their time. Overall, 1408 tasks (18.3%) involved multitasking, for example, updating the electronic health record while talking to a colleague about a patient on the phone (data not shown). Tasks involving multitasking represented 5.5% (13 h 5 min) of total observation time.

**Table 2.** GPs’ work activities recorded in time–motion data over 238 hours of practice in 61 clinical sessions

**Task category**	**Tasks, *n***	**Proportion of all tasks (95% CI)**	**Category-specific task time^a^**	**Proportion of total observed time (95% CI)**	**Tasks in category disrupted by operational failures, *n***	**Proportion of operational failures that disrupted a task in this category (95% CI)**
Direct care	6214	80.9 (80.0 to 81.8)	172:24:14	72.4 (70.2 to 74.6)	259	68.0 (63.3 to 72.7)
Clinical paperwork	767	10.0 (9.3 to 10.7)	30:32:52	12.8 (11.4 to 14.2)	57	15.0 (11.4 to 18.6)
Interactions	280	3.6 (3.2 to 4.1)	28:19:37	11.9 (9.7 to 14.1)	40	10.5 (7.4 to 13.6)
Internet use	103	1.3 (1.1 to 1.6)	2:18:08	1.0 (0.7 to 1.2)	9	2.4 (0.9 to 3.9)
Operational failures	381	5.0 (4.5 to 5.4)	9:12:18	3.9 (3.2 to 4.5)	16	4.2 (2.2 to 6.2)
Home visits	4	0.1 (0.0 to 0.1)	2:48:33	1.2 (−0.2 to 2.5)	0	0
Total	7749^b^	100.9^b^	245:35:42^c^	103.2^c^	381	100.1

a *Recorded in hours, minutes, seconds.*

b *Total number exceeds total tasks observed (*n *= 7679) as some tasks included components of* >*2 main categories.*

c *Total time exceeds total observation time (238 hours) because of multitasking involving* >*2 main categories.*

Using the pre-programmed categories developed by this study group, 381 operational failures were recorded, corresponding to 5.0% (95% CI = 4.5% to 5.4%) of total tasks (average 1.6 failures per GP per hour; examples in Table 3). The number of failures recorded during a session was significantly associated with duty-doctor sessions (<0.001), higher numbers of GPs (<0.001), and registered patients in a practice (<0.001) (data not shown). There was no association between the number of failures and GPs’ sex, practice alliances, or type of electronic health record. The tasks most frequently disrupted by operational failures were direct patient care (68.0%, *n* = 259, 95% CI = 63.3% to 72.7%), followed by clinical paperwork (15.0%, *n* = 57, 95% CI = 11.4% to 18.6%) (Table 2).

**Table 3.** Types of operational failure captured in the time-motion data

**The category of failure**	**Instances, *n***	**Proportion of total operational failures (95% CI)**	**Time consumed^a^**	**Proportion of total time consumed by operational failures (95% CI)**
Interruption due to other staff entering consultation room	114	29.8 (25.2 to 34.3)	2:37:16	28.4 (23.9 to 33.0)
Interruption due to other interactions with colleagues about patients	74	19.3 (15.4 to 23.3)	1:38:37	17.8 (14.0 to 21.7)
Interruption due to incoming work-related phone calls	43	11.2 (8.1 to 14.4)	1:10:51	12.8 (9.5 to 16.2)
Missing equipment or supplies	36	9.4 (6.5 to 12.3)	0:37:07	6.7 (4.2 to 9.2)
Problems with computers, technology, electronic health record	33	8.6 (5.8 to 11.4)	1:18:18	14.2 (10.7 to 17.7)
Interruption with request for paperwork: prescribing, reading/writing letters, actioning tasks	29	7.6 (4.9 to 10.2)	0:21:58	4.0 (2.0 to 5.9)
Interruption due to interactions with colleagues about processes	23	6.0 (3.6 to 8.4)	0:26:44	4.8 (2.7 to 7.0)
Other unclassified interruptions to the consultation	23	6.0 (3.6 to 8.4)	0:55:35	10.1 (7.0 to 13.1)
Interruptions due to personal interactions	6	1.6 (0.3 to 1.2)	0:05:25	1.0 (0.0 to 2.0)
Teaching-related interruptions^b^	2	0.5 (−0.2 to 1.2)	0:00:58	0.2 (−0.2 to 0.6)
Total	383^c^	100	9:12:49	100

a *Recorded in hours, minutes, seconds.*

b *Teaching-related interruptions relate to GP registrars or medical students interrupting a GP mid-consultation for help with a patient they were seeing themselves.*

c *Exceeds the number of tasks for operational failures and interruptions (*n *= 381) in* Table 2 because of some instances including >*2 subcategories.*

Unexpected interruptions to GPs’ work by practice colleagues, external individuals, or electronic requests seeking immediate response (Table 3) accounted for the majority (*n* = 306, 79.9%, 95% CI = 75.9% to 83.9%) of operational failures. Missing equipment or supplies represented 9.4% (*n* = 36, 95% CI = 6.5% to 12.3%) and were the next most frequent category, consuming 6.7% (37 min 7 s) of time spent by GPs dealing with failures overall. Problems with computers, technology, and electronic health records represented 8.6% (*n* = 33, 95% CI = 5.8% to 11.4%) of the failures recorded in the time–motion data, but consumed 14.2% (1 h 18 min 18 s) of time spent dealing with failures.

Analysis of the qualitative data — ethnography and interviews — identified 745 operational failures, 201 of which were unique to the qualitative study and not captured by the pre-programmed categories, suggesting that a large number of operational failures were missed by the time–motion method.

### Nature of operational failures and their impact

Synthesising the information from the time–motion study with the ethnographic and interview data, four major types of operational failures affecting GPs and their impact were characterised. The first two — interruptions and problems in equipment and supplies — were already evident in the time–motion data. Two further major types of operational failure — challenges linked to GPs’ coordination role, and practice work system and process problems (see Box 2 for examples) — were identified from the ethnographic and interview data. The four categories shared some features: for example, they all interfered with task completion and created extra work for GPs, but they varied in many other regards, including aetiology, the level of frustration caused, ability to work around or compensate for the issue, and the level of control GPs had over preventing recurrence.

**Box 2.** Categories of operational failure based on synthesis of qualitative (ethnographic fieldnotes and interviews) and quantitative (time-motion) data

**Category**	**Definition**	**Examples**	**Quotes**
**Interruptions**	Unexpected suspension of a GPs’ work task because an individual or device is seeking an immediate response from the GP	Interruptions to consultation by electronic messages, task alerts, and computer pop-ups; staff entering GPs consultation room with prescriptions for signing, requests to review a patient, or queries about practice management issues; phone calls into consultations from reception or external healthcare professionals; and teaching-related or personal interruptions	*‘The GP got a message from one of the nurses in the practice, who wanted to give a flu jab to somebody, but they couldn’t give this flu jab until the doctor had done a patient-specific direction form. So, the doctor quickly accessed the patient details, signed the form and sent it back to the nurse.’* (GP_H5_observation) *‘The instant messages are a real distraction from what you’re doing. People seem to think instant message means instant reply and actually well, no I’m busy at the moment … I think they’re possibly the most distracting because they’re there but they’re not there. The patient doesn’t really know about them. Like a phone call or somebody walking in, the patient at least knows there’s an interruption. But the instant messages, the patient doesn’t realise why you’re suddenly distracted.’* (GP_S3_interview)
**Problems in the availability of supplies and function of equipment**	Disruption or error in the availability of supplies or function of equipment, supplies needed by a GP to complete a work task	Consultation room not stocked with needed supplies such as urine containers or lubricant; equipment such as baby weighing scales or thermometers going missing; computer freezing or crashing; and problems with function of and information within the electronic health record	*‘He updated the electronic health record for the lady who had got the low blood pressure. He typed in quite a lot, then clicked on save but actually found that he couldn’t save it and a thing came up that said you are attempting to save a safeguarding issue and it was like what’s that I’ve never seen that before? And it wouldn’t let him save so he couldn’t progress any further.’* (GP_S4_observation) *‘The doctor asked if she could do a urine sample and went to get one of the specimen pots but there wasn’t any there. So, he had to go outside to one of the other rooms, and got some more pots. He explained that they used to have ancillary staff make sure that all the shelves were stocked up with everything that they needed, such as urine bottles, ear tips, tongue depressors etc, but they don’t have that anymore, and he said that’s quite infuriating. Maybe he should have checked at the beginning of the session, but he’d been really busy, he’d been to a visit, he’d had two emergency patients pushed in really quickly, so he hadn’t had time. So he said that’s one of the things that frustrates him.’* (GP_F4_observation)
**Operational failures related to GPs’ coordination role**	Disruptions to GPs’ work arising from problems in coordinating the care of patients	Issues with incorrect, delayed, insufficient, or missing information from external healthcare teams; problems referring patients into different healthcare services; and issues caused by external teams not following up on or requesting indicated tests, not arranging follow-up, or not providing information to the patient	*‘She said normally we’ll get a follow-up letter from the hospital, but that doesn’t seem to have happened, so, she would look into it, she would write to the hospital.’* (GP_C2_observation) *‘She said we’ve not got your discharge letter yet, so we can’t really see what they are going to do at the moment, but hopefully in four weeks’ time when she saw him again, they would be able to talk about it.’* (GP_F8_observation) *‘The people at the hospital have asked the GP to order an MRI* [magnetic resonance image], *and the GP says he finds that really crazy, that there’s something about the hospital system which isn’t working when it sends patients back to them unnecessarily to try and get them to do things which actually they can’t do.’* (GP_P6_observation)
**Operational failures arising from problems in practice processes**	Processes in the practice that are not fit for purpose, poorly documented, or out of date, resulting in duplication of work, inefficiency, or waste	Discrepancies in information provided to patients by different staff; problems in organisation of blood tests because of a lack of internal standard operating procedures; problems in the allocation of work within the practice; insufficient time allocated to specific tasks leading to multitasking and stress; and inefficiencies and discontinuity in allocation of information within practice	*‘He explained that the list of appointments is not necessarily truly reflective of the patients they talk to or see during the day.’* (GP_B3_observation) *‘The patient wanted the GP to set up the system so that she could make appointments for her kids. The GP sent her downstairs to the receptionist and he thought that was it. Then during the next appointment, the reception called and interrupted the consultation and said “we can’t do this, you have to do it yourself”. He said no, I can’t, and they said, yes, you need to. He tried to do it but called again the reception, to say, well, I can’t do it for this and that reasons.’* (GP_B4_observation) *‘There’s been problems in the past where the GP had tasked admin team about a blood test for a patient but the wrong test had been requested. So she said that in order to get the right test requested, she’d rather do it.’* (GP_F3_observation)

### Interruptions

Interruptions generally involved staff seeking input into a patient’s care via a knock on the GPs’ door (for example, to review clinical signs or symptoms, sign a prescription, or review an electrocardiograph) or a phone call:

*‘She went out to get the patient but she was interrupted … somebody from the admin team came in with a prescription for a baby.’*

(GP_C2_observation)

Intrusions in the form of task requests and instant messages through the electronic health record were a common source of distraction. These requests, concerning matters of varying urgency, popped to the front of the GPs’ computer screen, preventing them from doing other work until they had interacted with the message. GPs had little control over these electronic intrusions, but found them disruptive:

*‘One of the things that he found particularly draining and frustrating and a major interruption was the pop-up messages that they get constantly throughout the day, where it’s a task that comes in or a prescription that comes in. He said they just come in the centre of the screen.’*

(GP_O1_observation)

Numerous interruptions were associated with GPs’ non-clinical responsibilities, including, for example, those related to practice management or leadership of practice networks. These were dealt with during sessions and in-between patients:

*‘He had a lot of business around the practice network throughout the day; he was answering telephone calls, answering texts and also at one point going out of the room during a patient consultation to talk to somebody.’*

(GP_J1_observation)

GPs were more tolerant and non-judgemental of interruptions by practice staff or calls from external healthcare professionals than they were of other operational failures, seeing many goal-driven interruptions related to patient care as important to safe and effective functioning:

*‘I’d much rather that one of the nurses comes and grabs me rather than asks the patient to re-book, and I think over time it just builds confidence and a knowledge base.’*

(GP_P3_ interview)

However, it was also evident that many interruptions, even when goal-driven, were not time critical and appeared not always to be warranted. Although interruptions served an immediate purpose for the interrupter, they often left the interrupted GPs struggling to refocus and resume their original task. As well as threatening efficiency, interruptions could introduce risks associated with divided cognition and attention, which were sometimes safety-critical:

*‘The GP said that interruptions for signing prescriptions can be fine if the patient they’re seeing is a simple case, but it’s quite disruptive when they’re in complex consultation or with patients with whom it’s difficult to interact.’*

(GP_W5_observation)

### Problems relating to equipment and supplies

A second category of operational failure related to the physical objects needed by GPs to do their work. GPs usually required only a narrow repertoire of equipment and supplies, but when they were absent or malfunctioning the effects were disruptive.

#### Unstocked supplies and missing equipment

Stocking of consultation rooms was variably implemented between practices. Unstocked supplies, including otoscope and thermometer covers, urine containers, couch roll, lubricants, hand gel, and speculums were frequent.

Equipment such as baby scales and pulse oximeters were susceptible to going missing. These operational failures were usually (although not always) resolved in real time by the GP searching for the item needed. Such efforts, which the current author group has previously called *compensatory labour*,^19^ were a source of stress for GPs, causing multiple knock-on effects including protracted consultations, running late for other patients, and sometimes having to abandon a task:

*‘Some staple supplies like tongue depressors weren’t in the room. I know the solution is having the room standardised with all supplies, but that means taking a member of staff to do that. That is one of the bigger disruptions, because it means physically leaving the room, walking out, getting into the rooms with the supplies which are also locked.’*

(GP_B6_interview).

#### Problems with computers and technology

GPs routinely had to contend with frustratingly slow computers or crashing software, often resulting in cumulative lateness. Fluctuations in computer speeds led GPs to defer tasks like ordering X-rays or opening large patient files until after hours:

*‘Sometimes she would get through, get access to the blood tests form, and be ticking those boxes, then all of a sudden after she’d spent time doing that, the computer would freeze again. She often had to leave organising the blood tests until later, and said she would give any information to the receptionist, and they then had to come back in, the patients, to pick up any forms ... it was extremely frustrating.’*

(GP_H3_observation)

### Operational failures linked to GPs’ coordination role

A third set of operational failures arose from the distinctive role of the GP in coordinating patient care, where coordination is defined as *‘integrating or linking together different parts of an organisation to accomplish a collective set of tasks.’* ^30^ In this current study it was found that GPs operated at the nexus of a highly distributed, complex, and interdependent network of information, people, and institutions that had to be coordinated around each patient:

*‘This role has evolved to where we’re sort of like the ringleader to hold everything in the middle. We’re supposed to keep all the records, make sure everything is up to date, what their medication list is, make sure any referrals are done, etc.’*

(GP_H1_interview)

A striking finding was that although GPs had ultimate responsibility for coordinating the care given to patients, they had varying, and often no, control over many elements of the distributed network in which they had to operate. GPs oversaw the enactment of management plans as solo individuals, but crucially had to rely on, mediate, and bridge the boundaries between multiple forms of input into patients’ care:

*‘She said this is a really complex patient, and she gave a huge sigh. She didn’t really manage to resolve this problem by the end of it. She concluded that she was going to speak to the patient on the phone, write to the cardiologist and to the renal physician about what to do next.’*

(GP_C4_observation)

#### Missing information

Information failures, related to the supply of information from other healthcare services, were very disruptive and difficult to remedy immediately. Delayed, missing, and ambiguous discharge letters were especially troublesome. GPs took multiple compensatory actions: writing letters, calling other services, or working with practice colleagues to track down missing information, but usually were unable to solve the problem during the consultation. This led GPs to make temporising decisions and ask patients to re-attend:

*‘The patient had been discharged on a medication — there was no indication of the dose of medication or how long they need to be taking it for, or any details. She’d written to them to ask about this, and the consultant had written back, but there still wasn’t a comment on how long this patient should be on this medication for. The GP was quite annoyed. The patient hadn’t brought his medication with him — he had to go home, take a photograph of the medication so that she knew what he was taking.’*

(GP_F3_observation)

Consistent with their role in brokering between different services, this current study identified that GPs tended to intervene where gaps in communication occurred; for example, when patients did not receive external followup appointments, could not communicate with external healthcare providers between appointments, or did not understand what had happened at external appointments:

*‘They found a tumour and she’s supposed to have an operation but has not heard back from the hospital yet, so the patient came to the GP to get more information ... the GP did not know what’s happening. He said that they should have contacted them but as they haven’t he will try to sort out what’s going to happen.’*

(GP_P1_observation)

Their positioning as the main broker of patient care meant that GPs were also faced with large volumes of information to process, some of it *‘irrelevant and unnecessary’* (GP_ W2_interview), that compromised their ability to identify actionable items:

*‘The GP explained that letters from the hospital can be ten pages long — it’s really not clear where the important information is.’*

(GP_L5_observation)

#### Problems with arranging care and referrals for patients

Changes in what other services considered an appropriate referral were reported to be frequent. Referral forms often required patient information that the GP did not have to hand, thus demanding more time than was available in the consultation and delaying referral completion:

*‘This morning I had to refer a patient to hospital with a very mysterious illness … I had to make four phone calls to different places to try and … in the end she was presented to casualty. Often it’s quite difficult if you need something urgently from the hospital, they aren’t always going to be cooperative and it can be quite time-consuming.’*

(GP_B5_ interview)

### Problems relating to practice work systems

GPs’ ability to proceed efficiently was sometimes compromised by work systems, routines, and processes in their own practice that were not always well documented or designed, or had not responded to changes in the volume or character of work over time.

#### Problems in role allocation

Mundane tasks that consumed GPs’ time but were not clinical in nature were perceived as disruptive to clinical work. GPs agreed that much of the administrative work they undertook could be delegated. However, who could legitimately take on these tasks, and how clinical tasks could be differentiated from administrative ones, was not always straightforward. Thus, though re-allocating apparently mundane tasks might seem attractive, GPs reported that it could increase stress for other staff, threaten collegiality, or wastefully bounce tasks between staff before being resolved:

*‘He explained that whenever a referral is simple, he will try to do it himself to save time for secretaries and also because otherwise sometimes the task might end up being lost or not given the level of priority that he expected.’*

(GP_B4_observation)

This sense of responsibility was especially emphasised by some GPs who were practice partners:

*‘When you’re the partner, it’s* [patient care] *your responsibility. The buck stops with me. If* [name of salaried GP] *was taken ill tomorrow and couldn’t come into work, it’s me that has to come in ... When you take away that need for the responsibility, then people don’t have the interest in it in the same way.’*

(GP_C1_ interview)

#### Lack of standardisation of practice processes

Important internal processes, including phlebotomy, medication management, and allocation of incoming patient information or patient queries to GPs often lacked agreed protocols or standard operating procedures. Opaque processes led to duplication of work for practice staff, delays in task completion, and discrepancies between staff in the advice given to patients. There were some indications that problems in information and relational discontinuity were more evident in larger practices or in practices with multiple part-time GPs.

Discontinuity of care was problematic because of how it reduced efficiency in consultations, for example, because GPs who were unfamiliar with a patient had to spend more time recapping on patients’ clinical details:

*‘The GP receives a lot of tasks in her inbox for patients that are not her patients, so she has to find out who is the GP for that patient and forward the task to them.’*

(GP_ B5_ observation)

#### Gaps between the formal schedule and how GPs spent their time

An important finding was that the formal schedule of clinical sessions did not accommodate the realities of how GPs spent their time. As well as the workload of coordinating patients’ care, there were requests and demands that originated from an increasing number of external sources, much of which was fitted into the interstices of the GPs’ day.

Behind-the-scenes tasks could accumulate in large numbers; in one observation, a GP reported that over 100 tasks had come in by the end of their session:

*‘We tend not to allocate time to that—all of our timetable is appointments and consultations and that sort of thing. So when you get another thing to do, it’s just stuck on the end as something else to be managed. Which means often it’s squeezed in your “lunch break”, or it’s after surgery or whatever, or you might ring between patients if you have a slack minute.’*

(GP_H1_interview)

Insufficient time within the formal clinical schedule also led GPs to multitask, to rush tasks, or to be unable to give tasks enough attention, further increasing pressure and stress, and creating anxiety about error:

*‘We’re trying to make medical decisions, all the time, under this extreme pressure, and she said, things can just go wrong very easily and very quickly. She said, we’re looking from left to right the whole time. We’re constantly multi-tasking and trying to do things as quickly as we can, but not really having the time to do it. She said the pressure is extreme and that there is a high risk of making mistakes.’*

(GP_C2_observation)

## DISCUSSION

### Summary

To the authors’ knowledge, this study is one of the first to quantify, using time–motion methods, how English GPs spend their time in clinical sessions, establishing that operational failures account for at least 5% of all tasks undertaken by GPs and consume a minimum of 4% of their time. This is not a trivial proportion of a precious resource. However, it is also an underestimate, as it does not include work done outside programmed clinical sessions (for example, lunchtimes and after-hours), and far more operational failures were identified in the qualitative data than those measured using the pre-programmed time–motion categories.

Just as importantly, however, this study shows that the impact of operational failures goes well beyond unproductive diversion of time: the qualitative data shows that operational failures are highly consequential for GPs’ experience of work, causing stress, anxiety, and frustration, and impairing their relationships with patients. Further, clinic schedules, appointments systems, and processes only poorly accommodated the reality of what GPs do, resulting in a ‘fictive schedule’,^31^ cumulative burden, and compensatory efforts. Multitasking — which represented 5.5% of observation time and 18.3% of tasks recorded — was frequently employed by GPs to manage the fictive schedule, but was in itself a source of additional stress and pressure.

Although current approaches to addressing the NHS general practice workforce crisis include efforts to train and retain more GPs,^32^^,^^33^ promote GP resilience,^34^ and release capacity for clinical work,^35^^–^^38^ the findings of the current study suggest that better targeting of operational failures at the level of the healthcare system, and within practices themselves, might have rich potential for improving the working lives of GPs and the care they offer to patients.

### Strengths and limitations

Mixed methods^20^^,^^39^ were valuable in studying operational failures in a context where they have not been examined before to the authors’ knowledge. The time–motion data yielded detailed numerical data on a range of failures, but were inherently limited to those identified as a problem a priori *.* The qualitative findings, which revealed a broader range of operational failures and some reconceptualisation of the appropriate classification of operational failures in primary care, will enable future time–motion studies to be better informed. As in this current study it was not possible to conduct observations outside formally scheduled clinical sessions or during home visits when a significant proportion of GPs’ work — particularly around care coordination — occurs, the number of operational failures recorded was underestimated and capture of their full impact was limited. Extended observations outside formal office hours are needed.

This study focused on GPs; future work should examine operational failures as they affect other healthcare professionals and administrative staff in primary care, as well as patients themselves. It was conducted just before the COVID-19 pandemic, when most consultations were carried out face to face.

Future work should seek to replicate elements of this study in contexts where more care is being provided remotely. Observations took place in 28 general practices that were broadly similar to the national profile (Table 1), but may not be representative of all practices in the NHS, and, as the sample was recruited through the Clinical Research Network, may have been especially interested in quality improvement.^40^^,^^41^

### Comparison with existing literature

Many of the most disruptive failures identified in this study related to GPs’ role in the coordination of patient care. In contrast to hospitals, where teams looking after a patient may be known and visible to each other, are able to agree shared goals, and have relatively well-defined divisions of labour, GPs had to manage *ad hoc* assemblages of individuals and systems where many of the components were largely out of sight and external to their own organisation.

A collective of people, systems, and technologies contributes to problems of information discontinuity and coordination challenge, making it difficult to identify who is responsible for what, a situation known as the problem of many hands.^42^

Other operational failures were more internal to practices, and were broadly similar in character to those previously noted in secondary care.^13^^,^^14^^,^^16^^,^^43^^–^^49^ One important challenge for future research lies in distinguishing which interruptions are time critical and warrant instant access to GPs, and which could be addressed differently.^50^

Many failures arose from weaknesses in organisational work systems. Some of the solution may lie in organisational design — systematically *‘aligning structures, processes, leadership, culture, people, practices, and metrics to enable organisations to achieve their mission and strategy’*.^51^

The current study offers important directions for a future research agenda on operational failures in primary care. The number, growing size, and complexity of practices, often featuring new roles and varying the skill mix, warrants careful attention, including better understanding of relationships and skill mix within practice teams to inform workflow redesign and allocation of tasks that lie on the clinical–administrative divide.^52^^–^^54^

Frequently proposed solutions such as automation of administrative tasks through technology,^55^ role substitution, increasing the range of professionals in primary care, and other proposals for delegating tasks traditionally undertaken by GPs need to be handled with careful attention to work system and role design, and evaluated rigorously.^56^^,^^57^ Critically, improvement interventions should target the priorities of primary care teams and patients.

### Implications for research and practice

Progressive and equitable health care depends on high functioning primary care, to the extent that it is sometimes suggested that if general practice fails, the whole NHS fails.^2^ This study has identified that GPs experience frequent operational failures in their work, disrupting their ability to provide efficient patient care and imposing additional burdens in an already pressurised system. This improved understanding of the nature and impact of operational failures in primary care suggests important targets for improvement. It also indicates that support for addressing operational failures will be needed both at the level of the practice and wider health system.

At practice level, these findings indicate that investing time into the identification of operational failures experienced by individual GPs may lead to improvements in the safety, quality, and efficiency of patient care. At system level, improved mechanisms for coordinating care between general practice and other healthcare services are required if NHS priorities^58^ relating to the management of patients in community settings are to be realised. The pivotal role of the GP, and the need to optimise how information flows across boundaries and is actioned, will be key to this. Such improvements will depend on interdependent, integrated action with horizontal accountability and cooperation between all stakeholders. As integrated care systems^58^ continue to emerge, they should be sensitive to the challenges and vulnerabilities associated with GPs’ coordination role and the need to focus improvement efforts on directly supporting their work.

## Data

Numerical data are available on request to authors.
